# Stroboscopic neutron scattering measurements using Raspberry Pi-based sample environment event mode

**DOI:** 10.1107/S1600576726008332

**Published:** 2026-09-28

**Authors:** Andrew R. J. Nelson, Elliot P. Gilbert, Joshua P. King

**Affiliations:** a Australian Centre for Neutron Scattering, ANSTO, Locked Bag 2001, Kirrawee DC, NSW2232, Australia; Universität Duisburg-Essen, Germany

**Keywords:** stroboscopic measurements, data acquisition, kinetic measurements, event mode

## Abstract

An inexpensive single-board computer (Raspberry Pi 5) was employed to create event mode files from sample environment equipment, complementing event files from neutron scattering instrumentation. Together the sample and neutron event files enable fast kinetic processes to be studied via stroboscopic measurements. The device is adaptable to a broad array of experimental scenarios and demonstrates that cutting-edge neutron science can be achieved by employing inexpensive equipment with appropriate expertise.

## Abstract 2

An inexpensive device based on a Raspberry Pi can give neutron scattering instruments the ability to carry out stroboscopic measurements in a relatively simple and flexible fashion. Stroboscopic measurements can be used to study fast kinetic processes in condensed matter systems.

## 1. Introduction

Event mode is a form of data acquisition that many neutron scattering instruments are now able to employ, spanning both reactor (Smith *et al.*, 2012 ▸) and spallation (Peterson *et al.*, 2015 ▸; Granroth *et al.*, 2018 ▸; Adlmann *et al.*, 2015 ▸; Peterson *et al.*, 2018 ▸) sources. The characteristics of each neutron detection event are saved in a file, which can be post-processed to furnish information on kinetic processes occurring within the sample. Each event typically records the *X*/*Y* pixel position at which the neutron was detected, a frame number (*N*) and a time of flight (TOF). For instruments with pulsed neutron beams, such as at a spallation source, the frame number increments in line with the number of pulses travelling down the instrument. However, many steady-state instruments also operate with an internal framing source that is used to trigger the detector, including most of the instruments at the Australian Centre for Neutron Scattering. A neutron’s TOF denotes the elapsed time since the last frame pulse at which the neutron was detected. TOF information is used on energy-dispersive instruments to calculate a neutron’s wavelength. It is, however, also useful on monochromatic instruments, which possess their own intrinsic wavelength (and equivalently time) resolution. Here the frame number and TOF give a more precise arrival time, over and above the frame number alone.

Most kinetic experiments can be divided into two types, namely single shot and stroboscopic. In a single-shot experiment the kinetic process is started and multiple scattering patterns are acquired during the change, which gives sample information at various points along the reaction trajectory. Examples of single-shot experiments include thermal annealing (McEwan *et al.*, 2017 ▸; Clulow *et al.*, 2014 ▸), electrochemical deposition of thin films, charge/discharge of batteries (Sharma *et al.*, 2011 ▸; Ballantyne *et al.*, 2018 ▸), following chemical reaction kinetics (Merz *et al.*, 2023 ▸), temperature/magnetic field ramps (Heinze *et al.*, 2019 ▸) *etc*. Historically these experiments were conducted by configuring the data acquisition electronics (DAE) to collect consecutive measurements (time slices) of all the same duration. The major drawback of this approach is that the DAE must be set up ahead of the measurement, meaning the time-slice duration, and hence time resolution, must be selected in advance of knowing how fast the scattering patterns may change. In trajectory locations where the scattering patterns change slowly one can always add adjacent detector images together. However, if the scattering patterns change more quickly, the time resolution cannot be improved afterwards. The solution to this is event mode, where time-slice duration is adjusted during post-processing. Long durations can be used for slow change regions and *vice versa*. There is also no need to have time slices of equal duration. An example is the measurement of small-angle neutron scattering (SANS) alongside differential scanning calorimetry (DSC) on the Quokka SANS instrument at the Australian Centre for Neutron Scattering, where neutron data were binned with broad time resolution at temperatures below and above melting endotherms in a de-mixed binary polymer blend, and with much tighter resolution during the endotherm. This approach enabled structural changes to be directly correlated with thermal transitions in the system (Pullen *et al.*, 2014 ▸). The DSC is one of a range of unique sample environments that have been designed to operate on Quokka, including a temperature-controlled viscometer based on a Rapid ViscoAnalyser (Doutch *et al.*, 2012 ▸) and a device for heating and cooling a sample within the neutron beam at a rate of 10–20 K s^−1^ (Pullen *et al.*, 2008 ▸).

The ability to resolve kinetic changes in these single-shot experiments depends on how much the sample scatters, the difference between scattering patterns along the reaction trajectory and instrument flux. For Quokka a range of approaches are being actively explored to increase the already high flux, such as extended velocity tilt stage control (to broaden wavelength resolution) and white-beam optics. Both these approaches result in a greater timing uncertainty, which is of importance and further discussed below. Last, but not least, depending on the time resolution required, it is necessary to synchronize the scattering patterns with the time since the reaction trajectory started, as well as logging the sample environment (SE) information used to track reaction progress (*e.g.* temperature) at those points in time. This synchronization sometimes lacks sufficient precision and the time logging may not be frequent enough.

Stroboscopic experiments involve a stimulus that is applied repeatedly to a sample, measuring scattering patterns at different parts of a continuous stimulus wave (López-Barrón *et al.*, 2015 ▸) or at increasing elapsed times after a stimulus pulse (pump–probe) (Dalgliesh *et al.*, 2004 ▸; Porcar *et al.*, 2004*a* ▸; Porcar *et al.*, 2004*b* ▸). The timescale under examination is sufficiently short that data acquired during a subsection of a single stimulus are inevitably too noisy. Only by aggregating data from multiple applications of the stimulus does the statistical quality of the data become good enough for further analysis.

One example of a continuous stimulus wave is examination of colloidal structures undergoing large-amplitude oscillatory shear with small-angle scattering (López-Barrón *et al.*, 2012 ▸; López-Barrón *et al.*, 2015 ▸; Lee *et al.*, 2019 ▸), while pump–probe experiments include the relaxation of liquid crystal nematic phases at the solid–liquid interface after being oriented with an electric field (Dalgliesh *et al.*, 2004 ▸). Another example of a time-resolved stroboscopic measurement in the soft matter domain is a study by Wrede *et al.* (2018 ▸) using SANS to examine the volume phase transition kinetics of microgels being exposed to pressure jumps between 40 and 200 bar at 5 ms time resolution.

The TISANE approach (time-involved small-angle neutron experiment) pioneered by Gähler (Kipping *et al.*, 2008 ▸) is a stroboscopic approach that can access time resolutions down into the sub-millisecond regime (Bleuel, 2019 ▸). The setup requires a fast chopper, along with a detector with high time resolution and a sample environment in which the scattering properties of the sample are time modulated. Parameters for these modulations may be magnetic or electric fields, alternating currents, or ultrasonic pulses. A recent example has been reported in skyrmion kinetics (Mettus *et al.*, 2022 ▸).

Historically stroboscopic experiments were carried out either by the DAE frame pulse triggering the sample environment to start its stimulus (common on spallation sources) (Dalgliesh *et al.*, 2004 ▸) or by the sample environment triggering the DAE (the trigger is emitted at a given phase of the stimulus wave and acts as the framing pulse). In both approaches the DAE and sample environment timing configurations are set up to match each other, although the triggering ensures good synchronization. The DAE must also be configured in such a way that the scattering from the same stimulus location in multiple pulses accumulates in the same detector image/channel. A range of channels then represents the different phases of a continuous stimulus wave or, alternatively, differing elapsed times in a pump–probe experiment. During processing, corrections must be made for the sample-to-detector TOF, to avoid a systematic, wavelength-dependent, phase offset between the neutron detection time and the sample environment signal.

The triggering of one by the other can lead to restrictions placed on accessible sample conditions. For example, instruments using a pulsed neutron beam have a constant frame rate, so one is limited to choosing stimuli that are consistent with a multiple of the frame period. Stimulus frequencies much greater than the frame rate may be forbidden as synchronization of the SE with the frame source becomes difficult. Furthermore, the mutual dependence of the DAE and sample environment configurations can lead to a heavy experimental workload if there are a range of conditions that need to be examined. For example, in an oscillatory shear experiment one might want to measure at a range of frequencies, each one requiring a different DAE configuration, thereby making it challenging to automate; this is particularly relevant when conducting measurements during an ‘overnight run’.

A solution to the aforementioned challenges with single-shot and stroboscopic experiments is outlined here, namely event mode for sample environment. Here sample environment information is recorded one or more times per frame, creating a sample event file (SEF) that is a direct complement to the neutron event file (NEF). The information can be either a direct sensor reading of temperature/magnetic field, a voltage reading that is a proxy for sample condition (*e.g.* some rheometers have an output signal whose value corresponds to a velocity/shear rate *etc*.) or the positions of a slewing motor. The monitoring of continuous instrument movement is often used on synchrotron beamlines (Lippmann *et al.*, 2016 ▸), but here the focus is mainly on sample environment and knowing the state of the sample (environment) for each neutron that is detected. This SEF permits excellent synchronization between sample state information and associated scattering pattern for single-shot experiments. More importantly, for stroboscopic experiments, it allows the DAE to operate independently of the SE; there is no necessity for mutual configuration. Thus, when different SE conditions are employed, such as different stimulus frequencies, no reconfiguration is required. A wider range of sample conditions also becomes more accessible. One could, for example, choose the frame and stimulus frequencies to incorporate a large beat period to remove any aliasing effects on the acquired data. Moreover, stimulus frequencies higher than the frame rate are straightforward to measure (Adlmann *et al.*, 2015 ▸).

Sample event streaming has been available for at least a decade at the Spallation Neutron Source (SNS) [it is also used at ISIS and will be at the European Spallation Source (Mukai *et al.*, 2018 ▸)], where it has been integral to a wide range of hard and soft condensed matter systems (Granroth *et al.*, 2018 ▸). Important features of the SNS implementation are architecture that integrates the neutron and sample event streams into a single file using the widely adopted NeXus data format, a customized measurement board (‘ADCROC’) to measure and digitize sample environment information, and filtering of the event streams to provide scattering patterns for a given sample condition. Examples that utilize this combined neutron and sample event mode are diffraction studies of multiferroics in pulsed magnetic fields (Nojiri *et al.*, 2011 ▸), stroboscopic heating of supercooled high-temperature melts (Granroth *et al.*, 2018 ▸) and neutron reflectometry of concentrated surfactant micelles undergoing oscillatory shear at a solid–liquid interface (Adlmann *et al.*, 2015 ▸).

A variation on streaming a single type of value to the SEF during the single-shot or stroboscopic measurements could be to regularly record a spectrum, such as UV–Vis or IR absorption, alongside the NEF. For example, a sample environment was constructed to allow simultaneous UV–Vis absorption and SANS measurements on the TAIKAN instrument at the J-PARC Materials and Life Science Experimental Facility, which Iwase *et al.* (2023 ▸) used to study the structure of light-responsive micelles. Here, a time-sliced NEF follows the structure, with the UV–Vis being used to provide further insights into the mechanism. Whilst the spectroscopy was ‘acquired independently’ of SANS, it seems reasonable to assume that this would be a suitable system to study with combined NEF and SEF collection.

Reviews by Urban *et al.* (2021 ▸) and Mata *et al.* (2012 ▸) explore the use of time-resolved small-angle neutron scattering in soft condensed matter research, further illustrating the breadth of science that would benefit from combined NEF and SEF acquisition.

At the Australian Centre for Neutron Scattering (ACNS), the histogram memory server (HMS) and DAE have always been able to perform neutron event mode acquisition (Smith *et al.*, 2012 ▸); however, sample event streaming has been unavailable [the exception being a single measurement by Wolff *et al.* (2019 ▸)], although it is being built into the next generation DAE. In the interim, an inexpensive device (∼200 AUD) has been built from off-the-shelf items to add sample event streaming capability to most of the ACNS instruments. In this paper, the design and operation of the device is outlined, along with the first results from the monochromatic Quokka SANS (Wood *et al.*, 2018 ▸) instrument. We have also tested its operation with the time-of-flight Platypus neutron reflectometer (James *et al.*, 2011 ▸) (see supporting information).

## 2. Event streaming device (ESD)

The ESD is based on a 64-bit Raspberry Pi 5 (RPi) running Debian Bookworm with 8 GB of RAM. A schematic of the ESD is shown in Fig. 1 ▸.

The RPi has a 40 pin header for general purpose in/out (GPIO), allowing it to interface to a wide range of digital signals/devices.

### 2.1. MCP3008 eight-channel/10-bit analogue to digital converter (ADC)

The ADC converts a voltage coming from the sample environment, via a BNC connector, into a digital signal. The MCP3008 can measure up to eight channels at 10-bit resolution. When powered by a 3.3 V source it is only capable of measuring in the range 0–3.3 V. If the sample environment signal is wider (*e.g.* −10 to 10 V is used by Anton Paar rheometers), then appropriate shift and scale circuitry, such as a resistive divider, needs to be used to prevent damage to the ADC. Fig. 1 ▸ shows a resistive divider to scale a 0–10 V signal down to 0–3.1 V. A multimeter and oscilloscope are valuable diagnostic tools to prevent such damage. The ADC chip is capable of 200 kHz measurement.

### 2.2. uBlox NEO 7-M GPS board with external antenna

The RPi system clock is used to give each sample event an epoch timestamp. Epoch time is the number of seconds since 1 January 1970. To discipline the system clock, a GPS chip with an accurate pulse-per-second (PPS) and NMEA sentence output is used via the *pps-tools*, *gpsd* and *chrony* packages (Pivarnik, 2025 ▸). The system clock is therefore precise and stable to around a 1 µs level of magnitude (although, for typical neutron applications, even milliseconds would be acceptable). The NMEA sentences are read by the serial UART pins, with the PPS signal being directed to another pin. An external antenna is needed to receive GPS signal inside the neutron guide hall.

### 2.3. Framing signals

For normal operation a 5 V transistor–transistor logic (TTL) framing signal is used to trigger the Platypus and Quokka DAE. For Platypus this is emitted by its chopper system (24 Hz), whereas for Quokka this is normally an internal signal (50 Hz). This framing signal is used by the DAE to denote the start of the time of flight, commonly called *T*0 (‘t-zero’). When using the ESD, the Quokka HMS configuration is changed to use an external frame source, namely a signal generator outputting a 5 V pulse (2 ms long), typically running at 25 Hz. The framing signal is directed to both the DAE and the RPi; consequently there is no gradual phase drift of the *recording* of both streams. It is possible, however, that the sample environment signal, as supplied to the RPi, drifts in phase with respect to the framing signal. This is dealt with during data processing, as outlined below.

The framing signal is stepped down by a logic level converter as the maximum voltage on the RPi GPIO pins is 3.3 V. The GPIO pin is programmed to listen for rising edges, which are acted on by a software callback.

### 2.4. Histogram memory server (HMS)

The HMS server has a webserver front end that serves a text-based status page. The text status is a series of key:value fields. The fields relevant to ESD operation are listed in Table 1 ▸.

The *platypus_eventer* (*PE*) software on the RPi monitors the HMS status page on a 1 s basis. When the *DAQ* field indicates that a measurement is ‘*starting*/*started*’, the *PE* creates a data directory with the same name as *DAQ_dirname*, saving the status page into that directory. It then starts three processes that listen for rising edges on the frame signals, measure the ADC voltage several times during a frame and write data into the SEF. The first two processes send timestamped data to be written by the third process via a *queue*. The process listening for the frame signals emits a frame event when the *T*0 pulse arrives. When the process measuring ADC voltage identifies the frame event, it acknowledges the event and then conducts a measurement series with several voltages being recorded per frame. Whilst the ADC chip can measure at 200 kHz, a more reasonable sampling rate would be every 500 Hz, *i.e.* every ∼2 ms, to prevent the RPi from being overwhelmed. If, for example, it is determined that 20 values are required to characterize a sinusoidal waveform, at a 500 Hz ADC sample rate the upper limit for the sample environ­ment oscillation frequency in a stroboscopic experiment would be 25 Hz. Faster oscillation frequencies may be possible (50 Hz), but with an understanding that the ADC waveform is less precisely measured. Since the *PE* software is flexible it would be relatively simple for any of these processes to drive a GPIO pin high to interact with other equipment. This could be to give feedback to a sample environment or to trigger another piece of equipment (*e.g.* pump–probe every *N* frames, or initiating a spectrometer to record Fourier transform IR/UV–Vis data every *N* frames). When the *DAQ* state is no longer *started* these processes terminate and the SEF is closed.

Adapting the ESD for use at other facilities would require modification of the software so that the device knows when a measurement starts (first pulse) and stops. Often an instrument’s data acquisition electronics are able to emit logic signals for when a measurement is underway. Such an adaption is relatively straightforward; the software can be reprogrammed to listen and react to those logic signals on other GPIO pins. Alternatively, the ESD could be set up to continuously stream data in an approach akin to the Kafka streams used by the European Spallation Source (Mukai *et al.*, 2018 ▸), with the sample and neutron data being reconciled later. That may enable faster ADC sampling as the device is simplified; it need only provide timestamped voltage values to the Kafka stream and would not have the overhead of listening to the framing pulses.

### 2.5. Sample event file (SEF)

A fixed-size record is used for each event. The stream of records is saved to a compressed (gzip) file. The structure of each record is listed in Table 2 ▸, along with example entries. The width of the ‘voltage’ value (currently 2 bytes, float16) can be made larger if greater precision is required.

For a frame event a negative channel number is used and the voltage is NaN. For an ADC measurement the channel is positive and represents the ADC channel that was used for recording. The size of each record is 15 bytes. For an hour’s recording, at a frame frequency of 25 Hz and with one voltage recorded per frame, the SEF size is ∼1.4 MB; this size is negligible compared with the size of the NEF from a high-count-rate measurement.

Given the same frame signal triggers both the DAE and the ESD there should be a 1:1 correspondence between the NEF and SEF data. However, since the SEF stream is started before the NEF stream, the first step in post-processing is determining which SEF frame corresponds to the first NEF frame. The *PE* software does this by identifying which SEF frame corresponds most closely in time to *dataset_start_time_t*. The SEF frame number is thereby corrected by an appropriate offset. Verification of this offset calculation is explained in the supporting information.

The SEF data are also examined for glitches. These occur when two frame events arrive in rapid succession, or a frame event is missed. This could be due to noise or because the RPi is busy doing something else (it is not a real-time operating system). Identification of these glitches is carried out by calculating the difference between timestamps of adjacent frame events. If the difference is not close to the frame period, then a number of frames either side of the glitch are removed from the SEF. Subsequent frame numbers are then adjusted in an appropriate fashion. In practice these glitches rarely occur.

### 2.6. *platypus_eventer* software

The Python-based software to run the ESD and perform the initial processing of its event stream (*i.e.* read/process/deglitch/synchronize with NEF) is available at https://github.com/andyfaff/platypus_eventer with a BSD three-clause licence. The code required to read the NEF is present in the *refnx* package (Nelson & Prescott, 2019 ▸). Once the NEF and SEF have been synchronized, any further processing is then dependent on the type of experiment that was run; pump–probe, stroboscopic, single-shot and instrument slewing all require a different approach. However, a general principle still applies: filter and aggregate all neutron events that correspond to a given sample condition. Whilst filtering, the fraction of total acquisition time assigned to each scattering pattern is tracked; this fraction is used to scale beam monitor counts, detector time *etc*. The aggregated neutron events constitute a scattering pattern that is then processed by the normal instrument data reduction. We now proceed to describe how sample event mode can be used on Quokka, illustrated for a colloidal system undergoing large-amplitude oscillatory shear, and the associated processing steps involved.

## 3. Example – large-amplitude oscillatory shear (LAOS) with small-angle scattering

LAOS coupled with SANS is a technique for resolving how colloidal structures change in response to large nonlinear flow deformations. Experiments of this kind are carried out in a Couette geometry, using a rheometer mounted on a SANS instrument. Various groups have performed time-resolved (stroboscopic) LAOS-SANS measurements; two examples are the melting and recrystallization of micellar crystals (López-Barrón *et al.*, 2015 ▸) and identifying the molecular origins of the dynamic response of wormlike micelles (Rogers *et al.*, 2012 ▸). However, both of these examples involved the rheometer triggering the DAE, to allow the DAE to fill the different detector images in-modulo once per cycle. Here we use sample event streaming to carry out this kind of experiment.

### 3.1. Experiment description

The system of interest is a viscoelastic wormlike micelle (WLM) material composed of 158 m*M* cetylpyridinium chloride (CPCl), 62 m*M* sodium salicylate (NaSal) and 500 m*M* sodium chloride (NaCl) in D_2_O. WLMs are ubiquitous in consumer products, with their self-assembled, threadlike architecture giving rise to classical Maxwellian viscoelastic behaviour at sufficient concentrations (Dreiss & Feng, 2017 ▸; Gurnon *et al.*, 2014 ▸). Such rheological behaviour can be characterized from a frequency sweep conducted in the linear viscoelastic regime by the plateau in the elastic modulus, *G*_0_, and a single relaxation time, 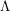, as shown in Fig. 2 ▸(*b*). In material processing during product manufacture or during end use application, WLM materials will undergo rapidly changing, large deformations that affect the structure and therefore the macroscale properties that determine how the material performs. These scenarios can be studied in a controlled setting using LAOS, with the structural response of the material recorded by SANS (Fig. 3).

An Anton Paar MCR302e rheometer was mounted onto the Quokka sample stage [Fig. 2 ▸(*a*)] and aligned to the velocity–vorticity plane (or 1–3 plane). The DAE and ESD used a 25 Hz framing signal created by a signal generator. The Anton Paar *RheoCompass* software was configured with the spindle angular deflection, ϕ, as an analogue voltage output that is fed to the ESD. Shear experiments were carried out at a frequency of *f* = 0.1 or 1 Hz (angular frequency ω = 0.628 or 6.28 rad s^−1^), at a shear strain amplitude of 100 or 1000% (γ_0_ = 1 or 10). The angular deflection is directly proportional to the shear strain, which is a sinusoidal function of time:
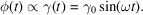


Shear strain can be derived from the deflection angle recorded by the ESD using the equation above since the two are in phase and the shear strain amplitude is a known input.

In the current implementation the shear stress was recorded and processed separately with the rheometer. By design, further inputs can be added to the ESD, which would allow for further rheological data to be recorded in the event stream.

To obtain the scattering patterns at each point in the sinusoidal oscillation, the NEF and SEF data are processed using a Python script (see supporting information). A brief description of the processing steps follows:

(1) The NeXus HDF (NX), NEF and SEF are retrieved. The NX file is queried for important parameters, such as sample-to-detector distance and nominal wavelength.

(2) The SEF is loaded, deglitched and frame-synchronized with the NEF.

(3) The fractional frame number of each voltage measurement is calculated. For example, if a voltage measurement is performed 5 ms into a 40 ms-long frame, *N*, then the fractional frame number is *N* + 0.125.

(4) The waveform of voltage (*V*) versus fractional frame number (*x*) is fitted with a four-parameter sinusoid:
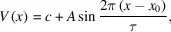
where *c* and *A* are an offset and amplitude. τ is the oscillation period of the rheometer expressed in frames (2 Hz rheometer oscillation with a 25 Hz frame signal would be τ = 12.5). *x*_0_ is a phase offset.

(5) TOF bins are created, based on a *subframe_bin_sz.* This value should divide exactly into the frame period. For a 40 ms period (25 Hz) a *subframe_bin_sz* = 2 ms is used. Even though Quokka is a monochromatic instrument, the TOF information recorded in the NEF can be used to improve the time resolution of the experiment beyond the period dictated by the frame frequency, down to the level specified by *subframe_bin_sz*. For this experiment we used a bin size of 2 ms. The exact time resolution available by this approach is controlled by the instrument’s wavelength resolution and sample-to-detector distance (SDD). The wavelength resolution on a monochromatic SANS instrument is determined by the settings of the neutron velocity selector (NVS), namely rotor speed and tilt angle, as well as the intrinsic blade design and geometry. On Quokka, the ASTRIUM-designed selector is typically operated with a tilt angle of zero degrees, yielding a full width at half-maximum wavelength resolution of 10%, *i.e.* Δλ/λ = 0.1. For the data in Fig. 3, a neutron wavelength of 6 Å was used; this corresponds to a neutron speed of 659.3 ms^−1^, the neutron taking 18.2 ms to reach the detector at a distance of 12 m from the sample. The corresponding timing uncertainty is therefore 1.82 ms. The most straightforward approach for improving time resolution would be to conduct measurements at shorter SDD; however, this is only acceptable if the relevant data occur in the higher *Q* region accessible in the configuration. An additional approach is to tilt the NVS in a positive direction, which has the effect of narrowing the wavelength resolution (a 10° tilt corresponds to 8.2% resolution). However, this causes the opposing effect of increasing wavelength and thus greater time uncertainty (at maximum selector speed, this corresponds to a wavelength of 7 Å). On Quokka the shortest standard wavelength is 4.5 Å, which is transmitted at the maximum rotor speed (28300 rpm). These neutrons have a speed of 879.1 ms^−1^ and arrive at a time of 1.48 ms for the shortest SDD of 1.3 m. The time uncertainty is then 0.15 ms for 10% resolution. An additional correction includes consideration of the scattering angle; larger scattering angles mean that neutrons travel an additional distance to reach the detector plane, by a factor of SDD/cos(2Θ). On Quokka, time resolutions on the order of 1 ms should be possible.

(6) A fractional frame number is assigned to each TOF bin. Assuming a *subframe_bin_sz* value of 2 ms, with a frame period of 40 ms, a neutron arriving in frame *N* with TOF = 3.141 ms would be digitized into bin 1, which spans [2, 4) ms. The midpoint of bin 1 is 3 ms, corresponding to a fractional frame number for that time bin of *N* + 3/40 = *N* + 0.075.

(7) The NEF is read and the TOF information for each neutron is digitized using the time bins specified in step (5). All neutrons therefore have a fractional frame number [step (6)].

(8) The fractional frame number for each neutron corresponds to the variable *x* from the equation given in step (4). The sample waveform phase, in radians, for each neutron is therefore given by
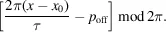
Here *p*_off_ is a correction for the neutron flight time, *i.e.* a 6 Å neutron arriving at the detector located 12 m downstream from the sample transited the sample environment 18.2 ms earlier. For a 1 Hz rheometer oscillation (period = 1000 ms) this corresponds to a phase correction of 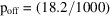
 × 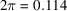
 radians.

(9) At this point each neutron event has an *X*/*Y* pixel position and a sample waveform phase. A [0, 2π) waveform is divided into NBINS parts. The neutron events are then digitized into the relevant NBINS intra-cycle detector image. For the data in Fig. 3 ▸ NBINS = 25.

(10) The number of time bins from all the frames, going into each intra-cycle image, is calculated; this is used to create a fraction that corresponds to the proportion of total collection time used to construct each image.

(11) The intra-cycle detector images [step (9)] and fractions [step (10)] are used to construct patched versions of the original NX file. Each patched file has its detector image overwritten, with the fractions being used to adjust beam monitor counts, acquisition times *etc*. This assumes that the beam monitor counts are distributed equally across the total measurement time. Here, each intra-cycle image corresponds to a total of 144 s.

(12) The patched NX files are then used in Quokka’s normal data reduction workflow (Kline, 2006 ▸).

It is possible for this processing to be limited to a subset of the data. For example, each hour of a multi-hour experiment can be considered separately, in case there are sample ageing effects.

In addition, steps (4)–(8) are carried out in a segmented fashion. The phase stability of the rheometer, or any sample environment, can vary over time, meaning that a single use of the equation in step (4) does not necessarily describe the waveform throughout the whole experiment. Here, it was found that processing data in 5 min segments worked well.

### 3.2. Results and discussion

The dimensionless Deborah (De) and Weissenberg (Wi) numbers can provide physical insight into the response of the WLM material to oscillatory shear conditions. The Deborah number is the ratio of the relaxation time (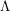 = 230 ms) of the material to the characteristic observation time. In this case we use the angular frequency, ω (the inverse of the characteristic observation time), to define the Deborah number: 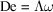. The Weissenberg number is given by 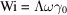, where 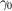 is the shear strain amplitude. A 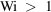 denotes the LAOS regime. As shown in Figs. 3 ▸(*a*)–3 ▸(*c*), various oscillatory shear conditions were explored, covering a range of De and Wi combinations. For each oscillation condition, SANS data were measured for a total of 3600 s.

The structural response of the WLM material is characterized using Hermans’ orientation parameter, 〈*P*2〉, which returns a value of zero for an isotropically arranged material, a value of 1 for a perfectly flow-aligned rod of infinitesimal thickness and −½ in the case of perfect perpendicular alignment. The calculation of 〈*P*2〉 from scattering patterns is described by Burger *et al.* (2010 ▸).

Fig. 3 ▸(*a*) is a control measurement in the small-amplitude oscillatory shear regime (De < 1, Wi < 1) that demonstrates that the material remains essentially isotropic under such conditions. For the LAOS conditions in Figs. 3 ▸(*b*) and 3 ▸(*c*), the orientation parameter loops as a function of shear strain; however, the position of minimum/maximum orientation depends on the De number (Table 3 ▸).

When De < 1 (*f* = 0.1 Hz), the material is expected to behave more viscously, which is consistent with Fig. 3 ▸(*b*) where the peak in the orientation parameter occurs near to the minimum shear strain (maximum shear rate). By contrast, when De > 1 (*f* = 1 Hz), the material exhibits more elastic-like deformation with greater levels of alignment near the maximum shear strain [Fig. 3 ▸(*c*)]. Representative 2D detector patterns processed using the NEF and SEF are shown in Fig. 3 ▸(*d*). These patterns correspond to the data in Fig. 3 ▸(*c*) and demonstrate an increasing level of anisotropy across a range of increasing shear strain.

## 4. Conclusions

It has been shown that sample event streaming can be achieved with a relatively simple setup to enable a variety of fast kinetic (including pump–probe, stroboscopic) measurements to be conducted routinely across neutron scattering instruments. Furthermore, the approach described can make single-shot experiments more robust. Variation of both the hardware and software to customize the device for different kinds of experiments is straightforward, resulting in extensive application. While a range of new experiments are already planned, *e.g.* magnetic response to varying field application, it is anticipated that this approach will stimulate a range of experimental scenarios that are not yet foreseen once communicated to the broader community of neutron instrument scientists and users.

## 5. Related literature

The following reference is cited only in the supporting information: Doucet *et al.* (2023 ▸). 

## Supplementary Material

Supporting information file. DOI: 10.1107/S1600576726008332/uz5037sup1.pdf

## Figures and Tables

**Figure 1:**
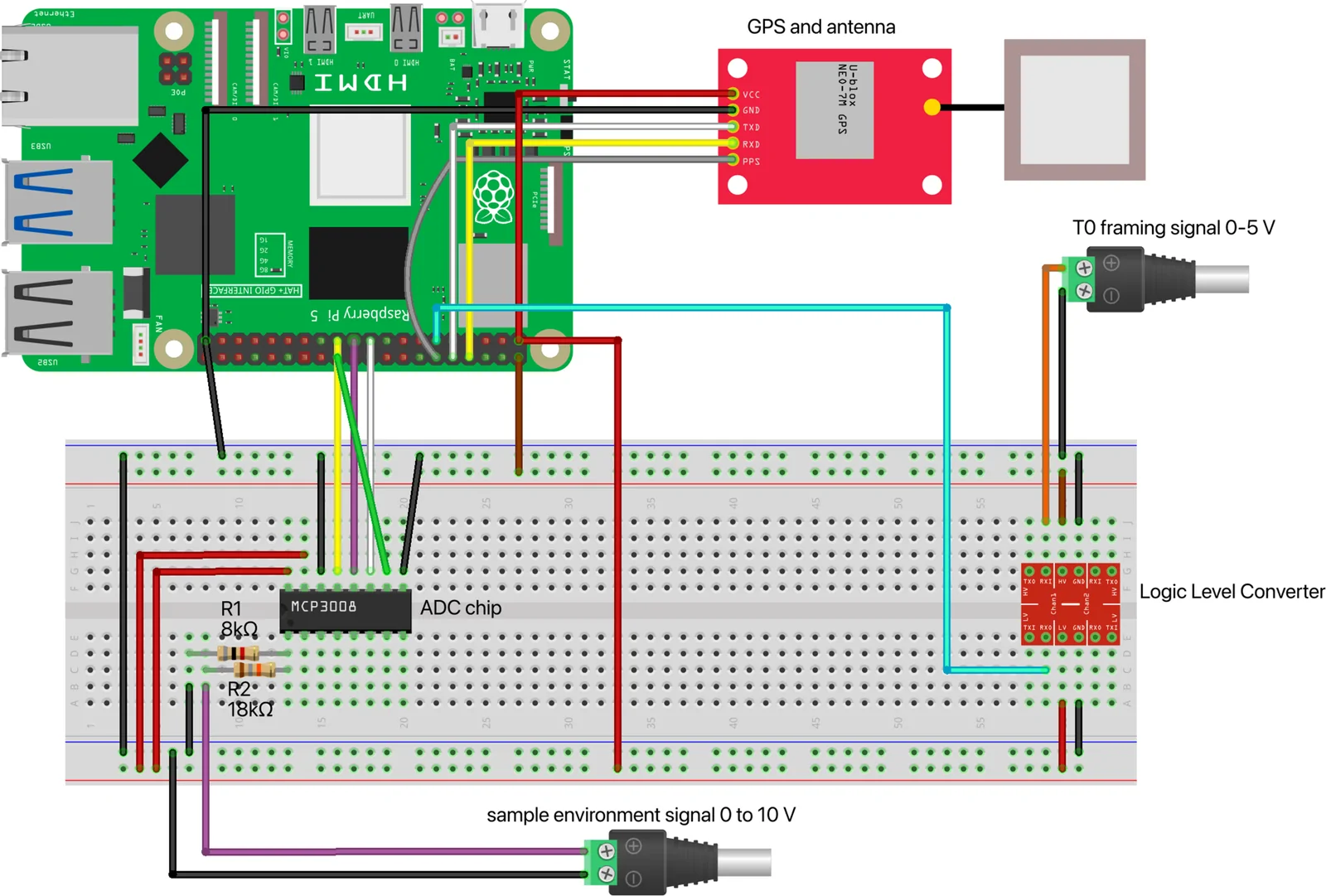
A wiring diagram of the ESD.

**Figure 2:**
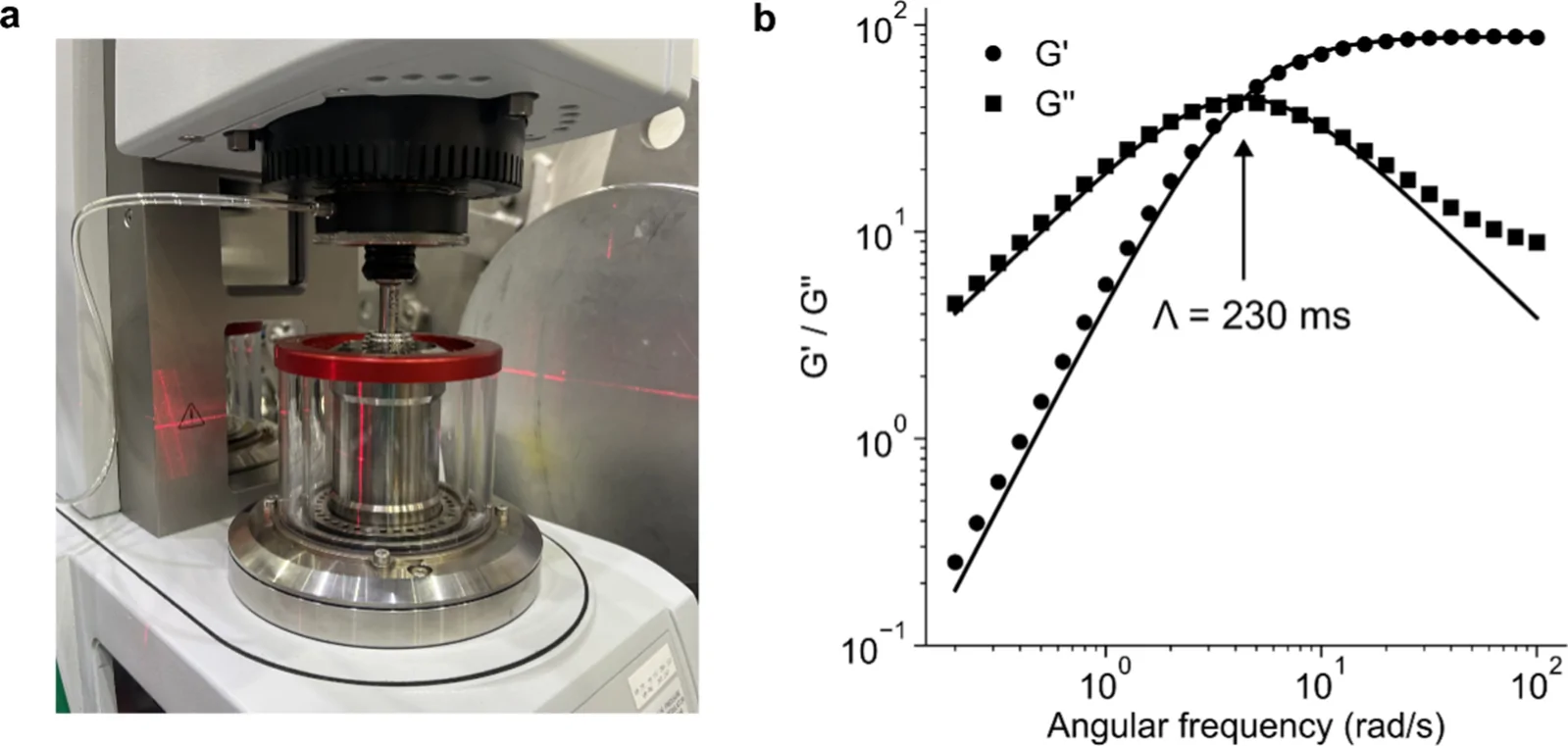
(*a*) MCR302e rheometer mounted on Quokka with the neutron beam aligned to the velocity–vorticity plane (1–3 plane). (*b*) Frequency sweep of a wormlike micelle material (158 m*M* CPCl, 62 m*M* NaSal, 500 m*M* NaCl in D_2_O) conducted within the linear viscoelastic regime with Maxwellian fit. The relaxation time, 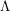, is indicated by the cross-over point of *G*′ and *G*′′.

**Figure 3:**
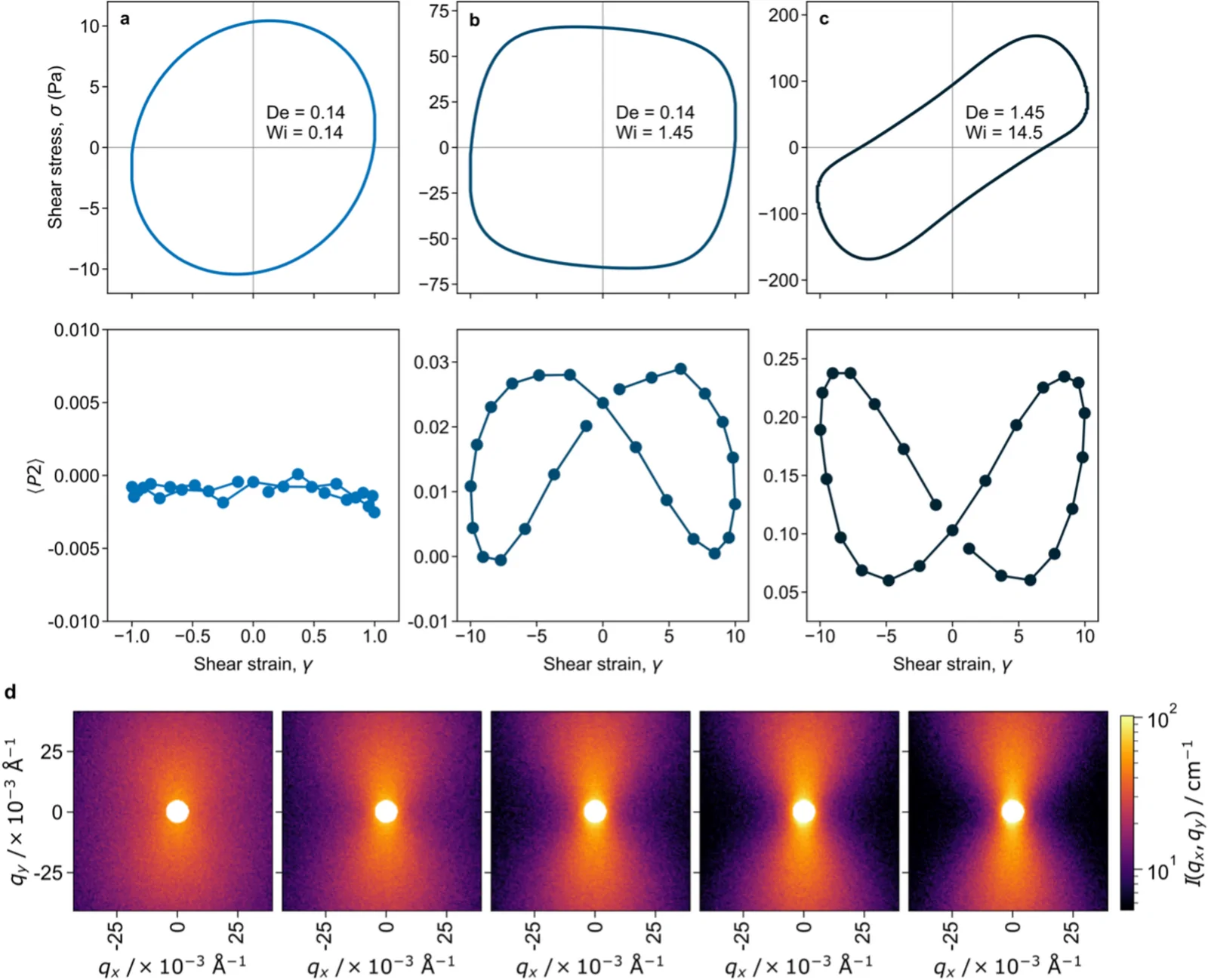
Time-resolved LAOS-SANS on a classical WLM material. (*a*–*c*) (Top) Elastic Lissajous–Bodwitch curves and (bottom) Hermans’ orientation parameter, 〈*P*2〉, for various oscillatory shear conditions as indicated by the Deborah (De) and Weissenberg (Wi) numbers. (*d*) Representative scattering patterns for the results shown in (*c*). See Table 3 ▸ for the shear strain and angular frequencies corresponding to the dimensionless De and Wi numbers.

**Table 1:** Important HMS status fields

Key	Value
*DAQ*	Whether the DAE state is *stopped*, *paused*, *starting*, *started*, *stopping*
*dataset_start_time_t*	Epoch time of when the first neutron frame was recorded down to microsecond level
*DAQ_dirname*	Name of a directory into which the NEF is saved (*e.g.* DAQ_2025-06-19T11-36-57)

**Table 2:** Structure of SEF records for a framing signal operating at 25 Hz

Frame No. (4 byte int)	Epoch time in ns (8 byte unsigned long long)	Channel (1 byte char)	Voltage (2 byte float)	Comment
0	1765580160308181000	−1	NaN	Channel of −1 denotes a frame event
0	1765580160309181000	1	1.5708	Voltage measurement on ADC pin 1
0	1765580160318181000	2	2.468	Voltage measurement on ADC pin 2
0	1765580160328181000	1	3.1416	Multiple voltage measurements are possible per frame; this entry is roughly halfway through the frame
1	1765580160348281000	−1	NaN	Next frame signal; should be approximately a frame period after the previous

**Table 3:** The shear strain and angular frequencies/frequencies corresponding to the dimensionless De and Wi numbers

Shear strain	Angular frequency (rad s^−1^)	Frequency (Hz)	De	Wi
1	0.63	0.1	0.14	0.14
10	0.63	0.1	0.14	1.45
10	6.3	1	1.45	14.5

## Data Availability

The *platypus_eventer* software may be obtained from https://github.com/andyfaff/platypus_eventer. The repository con­tains the script to operate the ESD, as well as the scripts for processing the SEF and NEF data presented in this paper.
