# Supplementary material for: Stroboscopic neutron scattering measurements using Raspberry Pi-based sample environment event mode
Source: J Appl Crystallogr. 2026 Sep 28;59(Pt 5):1645–54. doi: 10.1107/S1600576726008332 (PMC13636029; doi:10.1107/S1600576726008332)
Supplement: Supplementary file 1 [file j-59-01645-sup1.pdf]

JOURNAL OF APPLIED CRYSTALLOGRAPHY

Volume 59 (2026)

Supporting information for article:

Stroboscopic neutron scattering measurements using Raspberry Pi-based sample environment event mode

Andrew R. J. Nelson, Elliot P. Gilbert and Joshua P. King

S1. Verifying the synchronisation of the Neutron (NEF) and Sample Event (SEF) Files

The NEF and SEF files are synchronised by identifying which SEF frame has the closest epoch time to the *dataset_start_time_t* (Table 1) that is reported by the histogram memory server. The corresponding epoch times are typically within a few milliseconds, which is one order of magnitude smaller than the frame period. However, it was unclear as to whether the SEF frame with closest time correspondence, or the SEF frames on either side of it, should be considered the frame that is equivalent to the first NEF frame. To answer this question, a short experiment was carried out on the *Platypus* time-of-flight neutron reflectometer (James *et al.*, 2011). Platypus has four disk choppers, with a pair of them (1+2, 1+3, 1+4) rotating in an optically blind manner, at a typical frequency of 24 Hz. Here a third chopper was used, rotating at a quarter of the speed, i.e. choppers 1 and 3 were rotating at 24 Hz, with chopper 2 rotating in the same direction at 6 Hz. In such a setup only one frame in four is expected to have neutrons present, as the other three frames have chopper 2 blocking the beam. Small amendments were made to the hardware and software of the Event Streaming Device to record frame events for both chopper 1 (channel = -1) and chopper 2 (channel = -2) in the SEF. This information allows identification of which of the SEF frames for chopper 1 *also* had chopper 2 open. A quick check confirmed that after the chopper 1 frame in which chopper 2 was also open, the following three chopper 1 frames did not have any chopper 2 events.

A quick acquisition was then carried out with neutrons, with the NEF and SEF streams being synchronised by identifying the SEF frame whose epoch time was closest to *dataset_start_time_t*. The NEF file was processed to give the total number of neutrons per frame. As expected, there were only significant numbers of neutrons in every fourth frame. More importantly the NEF frames with a large of number of neutrons also corresponded to the SEF frames in which both chopper 1 *and* 2 were open. In other words, the synchronisation using closest epoch time works correctly. This process was repeated several times to confirm it was not a coincidence.

S2. Materials and methods

Cetylpyridinium chloride monohydrate (CPCl, >99% purity, Sigma-Aldrich), sodium salicylate (NaSal, 99.5% purity, Sigma-Aldrich), sodium chloride (NaCl, >99.5% purity, Merck) and D_2O (99.8% purity, Merck) were used as supplied.

Required amounts of CPCl and NaSal were weighed out into a sample vial. The required volume of 500 mM NaCl in D_2O was added to achieve a final concentration of 158 mM CPCl and 62 mM NaSal. The sample was sealed, vortexed to disperse the material and then placed in an oven at 40 ˚C for 10 days to allow the sample to fully equilibrate.

The sample was loaded into a Couette cell comprising an outer quartz cup (diameter = 50 mm) with an inner titanium bob (diameter = 48 mm) giving a gap size of 1 mm and a total neutron path length of 2 mm. Sample temperature was maintained at 25 °C using an Anton Paar convection temperature device with quartz shrouds. A solvent trap was installed to limit sample evaporation during the measurement. A frequency sweep was conducted within the linear viscoelastic regime at a constant shear strain of 10% across an angular frequency range from 0.2 to 200 rad/s. Custom python code was used to fit the Maxwell model simultaneously to the elastic (G') and viscous (G'') moduli as a function of angular frequency from the frequency sweep data. This returned a relaxation time, Λ = 230 ms, and plateau modulus, G_0 = 88 Pa. Dimensionless Deborah (De) and Weissenberg (Wi) numbers were calculated based on this relaxation time.

Small-angle neutron scattering (SANS) data was collected on the *Quokka* instrument (Wood *et al.*, 2018) using a sample-to-detector distance of 12 m, a neutron wavelength of 6 Å, and a wavelength resolution of 10 %. Raw scattering data for a given condition was processed into 25 frames using the NEF and SEF files and then reduced using standard *Quokka* macros (Kline, 2006). Empty cell, empty beam, and sample transmission measurements were used to put the scattering intensity onto an absolute scale.

Intensity as function of the azimuthal angle from an annular region averaged over a q-range from 0.008 to 0.02 $Å^{-1}$ was extracted using the batch slicing tool in SasView (V5.0.6) (Doucet *et al.*, 2023). Custom Matlab code was used to normalise annular extractions (intensity as a function of azimuthal angle) and fit a series of Legendre polynomials. Hermans' orientation parameter, <P2>, was derived from the second term in the Legendre polynomial fit. Further details can be found in Burger et al (Burger *et al.*, 2010).
